# Convolutional Neural Networks for Fully Automated Diagnosis of Cardiac Amyloidosis by Cardiac Magnetic Resonance Imaging

**DOI:** 10.3390/jpm11121268

**Published:** 2021-12-01

**Authors:** Asan Agibetov, Andreas Kammerlander, Franz Duca, Christian Nitsche, Matthias Koschutnik, Carolina Donà, Theresa-Marie Dachs, René Rettl, Alessa Stria, Lore Schrutka, Christina Binder, Johannes Kastner, Hermine Agis, Renate Kain, Michaela Auer-Grumbach, Matthias Samwald, Christian Hengstenberg, Georg Dorffner, Julia Mascherbauer, Diana Bonderman

**Affiliations:** 1 Institute of Artificial Intelligence, Medical University of Vienna, 1090 Vienna, Austria; asan.agibetov@meduniwien.ac.at (A.A.); alessa.stria@meduniwien.ac.at (A.S.); matthias.samwald@meduniwien.ac.at (M.S.); georg.dorffner@meduniwien.ac.at (G.D.); 2 Division of Cardiology, Medical University of Vienna, 1090 Vienna, Austria; andreas.kammerlander@meduniwien.ac.at (A.K.); franz.duca@meduniwien.ac.at (F.D.); christian.nitsche@meduniwien.ac.at (C.N.); matthias.koschutnik@meduniwien.ac.at (M.K.); carolina.dona@meduniwien.ac.at (C.D.); theresa-marie.dachs@meduniwien.ac.at (T.-M.D.); rene.rettl@meduniwien.ac.at (R.R.); lore.schrutka@meduniwien.ac.at (L.S.); christina.binder@meduniwien.ac.at (C.B.); johannes.kastner@meduniwien.ac.at (J.K.); christian.hengstenberg@meduniwien.ac.at (C.H.); julia.mascherbauer@meduniwien.ac.at (J.M.); 3 Division of Oncology, Medical University of Vienna, 1090 Vienna, Austria; hermine.agis@meduniwien.ac.at; 4 Department of Pathology, Medical University of Vienna, 1090 Vienna, Austria; renate.kain@meduniwien.ac.at; 5 Division of Orthopedics and Traumatology, Medical University of Vienna, 1090 Vienna, Austria; michaela.auer-grumbach@meduniwien.ac.at

**Keywords:** heart failure, cardiac amyloidosis, artificial intelligence, diagnostic ability

## Abstract

Aims: We tested the hypothesis that artificial intelligence (AI)-powered algorithms applied to cardiac magnetic resonance (CMR) images could be able to detect the potential patterns of cardiac amyloidosis (CA). Readers in CMR centers with a low volume of referrals for the detection of myocardial storage diseases or a low volume of CMRs, in general, may overlook CA. In light of the growing prevalence of the disease and emerging therapeutic options, there is an urgent need to avoid misdiagnoses. Methods and Results: Using CMR data from 502 patients (CA: *n* = 82), we trained convolutional neural networks (CNNs) to automatically diagnose patients with CA. We compared the diagnostic accuracy of different state-of-the-art deep learning techniques on common CMR imaging protocols in detecting imaging patterns associated with CA. As a result of a 10-fold cross-validated evaluation, the best-performing fine-tuned CNN achieved an average ROC AUC score of 0.96, resulting in a diagnostic accuracy of 94% sensitivity and 90% specificity. Conclusions: Applying AI to CMR to diagnose CA may set a remarkable milestone in an attempt to establish a fully computational diagnostic path for the diagnosis of CA, in order to support the complex diagnostic work-up requiring a profound knowledge of experts from different disciplines.

## 1. Introduction

Amyloidosis is a complex, multisystemic disease that is caused by the deposition of misfolded protein fragments in the extracellular space of tissues [1,2]. Cardiac amyloidosis (CA) is associated with substantial morbidity and mortality. The increased use of cardiac magnetic resonance imaging (CMR) in cardiology has revealed a previously unrecognized prevalence of CA, which has emerged from a “rare” disease that was often only diagnosed post mortem, to a condition of significant clinical relevance that every cardiologist is confronted with. An autopsy study could demonstrate the presence of CA in 25% of elderly people (≥85 years) [3]. Further studies showed that 14% of patients undergoing transcatheter aortic valve implantation, 13% of heart failure patients with preserved ejection fraction (HFpEF), and 8% of severe aortic stenosis patients suffer from concomitant CA [4,5,6].

The two predominant amyloid proteins found in the heart are transthyretin (TTR) and immunoglobulin light chains (AL). The expansion of the extracellular space due to amyloid deposition causes diastolic dysfunction of the left ventricle (LV). Eventually, affected patients develop severe heart failure (HF) and face a dismal prognosis [7].

A comprehensive algorithm for diagnostic work-up of CA has recently been published [8]. It includes CMR as one baseline diagnostic modality. However, the signs of CA may be unspecific in CMR scans, and CMR may even appear unremarkable although CA is present [9]. Furthermore, readers in CMR centers with a low volume of referrals for the detection of myocardial storage diseases or a low volume of cardiac CMRs in general may overlook nonspecific or rare signs of CA. In light of the high prevalence of the disease and emerging therapeutic options [10], we feel that there is an urgent need to avoid lacking CA diagnoses. We therefore used convolutional neural networks (CNNs) to develop a fully automated algorithm for the diagnosis of CA using CMR.

## 2. Methods

### 2.1. Study Population

We enrolled consecutive adult patients between August 2010 and August 2018 who underwent a complete CMR study at our tertiary care center at the Vienna General Hospital. Our center is located at the Medical University of Vienna and has a high-volume cardiac catheterization unit and a high-volume cardiac transplantation program. Moreover, we are part of the European Reference Network for Amyloidosis and a national referral center for patients with heart failure and preserved ejection fraction (HFpEF). Patients underwent clinical and laboratory assessment, electrocardiogram (ECG), transthoracic echocardiography, CMR, and, if any suspicion of CA was present, 99 mTc-DPD bone scintigraphy, as well as blood and urine tests for the detection of pathological light chains. The pre-CMR suspicion of CA was raised when patients presented with LV-hypertrophy, in particular those with interventricular septum thickness ≥15 mm and shortness of breath. In case of suspicion of AL-CA, myocardial biopsy was performed. In case of suspicion of TTR-CA, endomyocardial biopsy (EMB) was performed until 2016, when the paper by Gillmore et al. [8] on the diagnostic algorithm of CA was published. Thereafter, only AL-CA cases and TTR-CA with presence of monoclonal protein underwent myocardial biopsy. All patients provided written informed consent. The study was approved by the Ethics Committee of the Medical University of Vienna (EK no. 796/2010).

### 2.2. Imaging Protocols and Data Preparation

#### Cardiac Magnetic Resonance Imaging

CMR examinations were performed on a 1.5-T scanner (MAGNETOM Avanto; Siemens Healthcare GmbH, Erlangen, Germany), following standard protocols that included late gadolinium enhancement imaging (0.1 mmol/kg gadobutrol (Gadovist; Bayer Vital GmbH, Leverkusen, Germany)) if estimated glomerular filtration rate was ≥30 mL/min/1.73 m^2^ [11]. At the time of insertion of the intravenous cannula, blood was drawn for hematocrit and serum creatinine measurement. For analysis of late gadolinium enhancement (LGE) images, two independent reviewers judged whether a typical pattern for CA was present or not. Electrocardiographically triggered modified look-locker inversion recovery (MOLLI) using a 5(3)3 prototype (5 acquisition heartbeats followed by 3 recovery heartbeats and further 3 acquisition heartbeats) was applied for precontrast T1 mapping. This method generates an inline, pixel-based T1 map by acquiring a series of images over several heartbeats with shifted T1 times, inline motion correction, and inline calculation of the T1 relaxation curve within 1 breath hold. T1 sequence parameters were as follows: starting inversion time 120 ms, inversion time increment 80 ms, reconstructed matrix size 256 × 218, and measured matrix size 256 × 144 (phase-encoding resolution 66% and phase-encoding field of view 85%). T1 maps were created both before and 15 min after contrast agent application. For postcontrast T1 mapping, a 4(1)3(1)2 prototype was used. T1 values from a midcavity short-axis slice and a midcavity 4-chamber view were averaged for assessment of entire LV myocardium. For extracellular volume (ECV) calculation, the following formula was used [12]:

(1) $$\mathrm{MOLLI}-\mathrm{ECV}=\left(1-\mathrm{hematocrit}\right)\times \frac{\left(\frac{1}{T1\mathrm{myopost}}\right)-\left(\frac{1}{T1\mathrm{myopre}}\right)}{\left(\frac{1}{T1\mathrm{bloodpost}}\right)-\left(\frac{1}{T1\mathrm{bloodpre}}\right)}$$

T1 myo pre/T1 blood pre indicates myocardial/blood native T1 times and T1 myo post/T1 blood post indicates T1 times of myocardium/blood 15 min after gadobutrol application. The local reference range for normal MOLLI-ECV values is 25.4 ± 2.7%, derived from 36 healthy sex-matched controls [13].

The core CMR data set (*n* = 502) included patients with EMB-proven CA (*n* = 82, true positives) and 420 control patients with unrelated HF types (negative cases). In total, our CMR dataset contained 16,343 LGE (2598 positives; 13,745 negatives); 30,630 MOLLI (7649 positives; 28,032 negatives); and 309,702 CINE (53,878 positives; 255,824 negatives) images. For each patient, these three imaging protocols produced heterogeneous, in terms of size, sets of images.

### 2.3. Experimental Setting

To assess the performance of CNNs for fully automated CA diagnosis, we compared three different modeling techniques. We refer to them throughout this paper as: from scratch, feature extraction, and fine-tuning (Supplementary Figures S1–S3). From scratch is a standard deep convolutional pipeline based on progressive image downsampling [13]. Feature extraction and fine-tuning are two transfer learning techniques [14]. We used a pretrained VGG16 [15] CNN architecture on the ImageNet dataset for both pipelines. In case of feature extraction, last convolutional feature map activations of the pretrained VGG16 network were used as input features into a logistic regression classifier. For fine tuning, we retrained the four last convolutional layers of the pretrained VGG16 network, while keeping the weights of all other low-level layers intact. 

All our computational results were achieved on a 2 × Intel Xeon CPU server (12 cores each, base frequency 2.2 GHz) with 10 × NVIDIA GTX1080 TI GPU (11 GB GDDR5 each) and 8 × 32 Gb of RAM. For deep learning image classification pipelines, we used Keras Python library (version 2.1.6) with Tensorflow-GPU (version 1.8) as a backend for GPU utilization. Additionally, all our statistical and machine learning experiments were performed with the open-source Scikit-Learn Python package (version 0.20).

### 2.4. Data Preprocessing

For a fair comparison of all models, heart images were preprocessed with the same data preparation pipeline. All images corresponding to a specific imaging modality were extracted from DICOM files. To all images, we applied the following transformations: (i) histogram equalization to improve contrast, (ii) image resizing (target resolution 224 × 224) to standardize input for pretrained networks, and (iii) Gaussian smoothing to prevent aliasing due to downscaling. Moreover, each image was represented in three RGB channels (original grayscale images were duplicated three times for three channels). During training, we computed the mean pixel values for each channel of the training set and subtracted them from all images, both in training and validation datasets; in our case, mean pixel value for all the three channels was the same (per training set of images). These data preparation steps ensured all models to receive similarly prepared inputs, and therefore they facilitated a fair comparison of different methods.

### 2.5. Statistical Analysis of Convolutional Neural Network Performance

To assess model’s performance and its statistical variance, we employed a 10-fold cross-validation (CV) separately for each deep learning technique and imaging protocol. To prevent information leakage from training and validation, each CV fold of imaging data was split among patients—no overlapping patient images in training and validation sets. Moreover, splits were generated in a stratified fashion preserving class sample ratio. Since our classification problem is an imbalanced one, with more negative samples than positives, area under the receiver-operating characteristic curve (ROC AUC) score was chosen as our performance measure. For each averaged ROC curve, we also reported diagnostic accuracy in terms of sensitivity and specificity. These were determined by extracting operating points from the ROC curves. We used Youden’s J statistic [16] to determine optimal operating points. In all our experiments, we reported and compared two classification scores per model: image classification and patient classification. In case of image classification, each image was treated as an independent measurement, i.e., two images of the same patient were classified independently. For patient classification, averages of all patient image predictions were compared, and patient classification was treated as average voting.

To prevent overfitting and ensure model generalization in each CV fold, training data were further split into training and development sets in 80/20 ratio. All CNN models were then trained for a maximum of 1000 epochs on the reduced training set using stochastic gradient descent optimizer with momentum and weight decay [17], i.e., L2 regularization. Furthermore, the training process was regularized with early stopping [18] on the ROC AUC score on the development set training continued as long as the ROC AUC score on the development set kept improving. To ensure gradual parameter update for fine-tuned CNNs, we set the learning rate to 0.001; for from-scratch CNNs, the learning rate was set to 0.01.

Our experimental setting thus allowed fair and statistical comparison of all models considered. For all analyses, a *p*-value < 0.05 was considered statistically significant.

## 3. Results

### 3.1. Clinical Characteristics of Study Participants

The detailed clinical baseline characteristics for the 502 consecutively registered patients are displayed in Table 1. In brief, 82 (16.3%) were diagnosed with CA-associated HF (positive cases), and the remaining 420 with unrelated HF types (negative cases). Among the negatives, the predominant condition was HFpEF (*n* = 163), 107 patients were diagnosed with ischemic cardiomyopathy, 53 were diagnosed with hypertrophic and other cardiomyopathies, 44 patients had valvular heart disease, 30 patients suffered from cardiac sarcoidosis, 19 patients had HF condition linked with congenital heart disease, including muscular dystrophies, and the remaining 4 patients were diagnosed with rare HF conditions, such as pericardial disease (*n* = 3) and left atrial myxoma (*n* = 1). CA patients were predominantly male (65.8% of CA patients and 44.9% of controls, *p* = 0.003) and older (median age 75.0 years [68.0–82.5] vs. 66.0 years [50.0–75.0] in controls, *p* < 0.001). It is also important to note that CA-related HF patients were in rather advanced disease stages when compared to controls, as documented by higher NT-proBNP levels (median NT-proBNP in pg/mL: 3002.0 [1282.5, 7453.0] in CA patients vs. 452.0 [143.9, 1380.0] in non-CA patients, *p* < 0.001).

### 3.2. Cardiac Magnetic Resonance Imaging-Based Diagnostic Ability of the Convolutional Neural Network

In Table 2, we report average ROC AUC scores of a 10-fold cross-validation for image and patient classifications for all three imaging protocols and the three convolutional architectures. In what follows, we group these results according to the (1) respective imaging protocol, (2) deep learning technique, and (3) effect of using multiple images vs. a single image for CA prediction, and analyzed the effect on the diagnostic accuracy for each group.

### 3.3. The effect of Imaging Protocol on Diagnostic Accuracy

**Figure 1:**
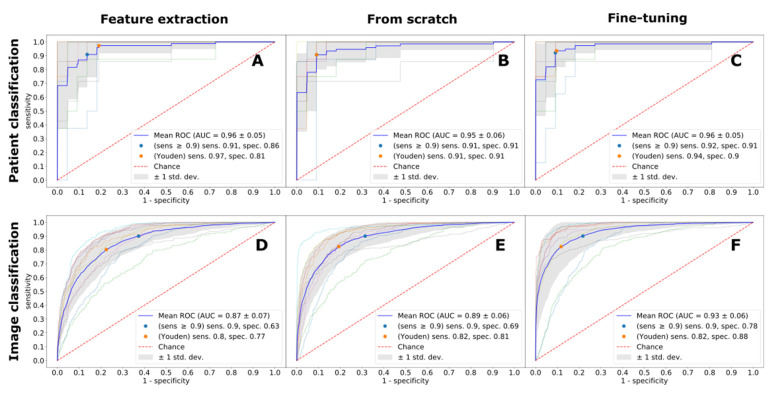
Image (panels (**A**–**C**)) and patient (panels (**D**–**F**)) classification performance by LGE images for all models measured with ROC curves. ROC AUC—Area under Receiver Operating Characteristic Curve, Sens.—Sensitivity, Spec.—Specificity.

**Figure 2:**
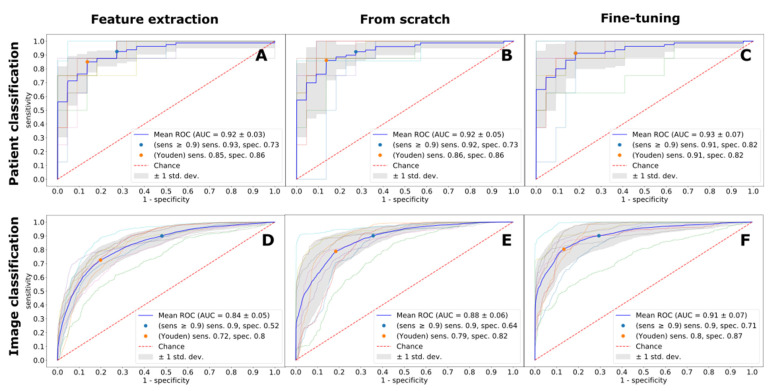
Image (panels (**A**–**C**)) and patient (panels (**D**–**F**)) classification performance by MOLLI images for all models measured with ROC curves. ROC AUC—Area under Receiver Operating Characteristic Curve, Sens.—Sensitivity, Spec.—Specificity.

**Figure 3:**
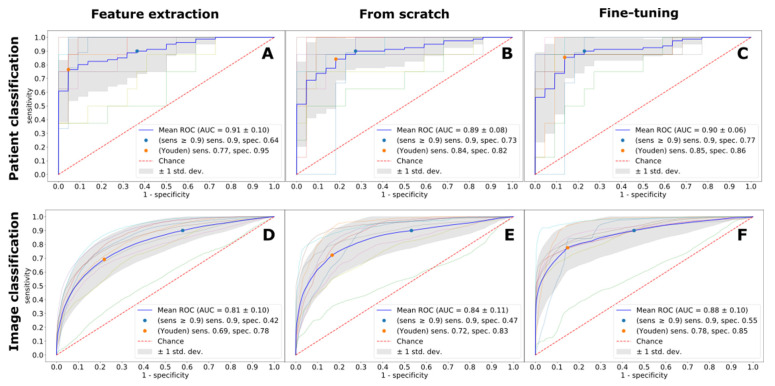
Image (panels (**A**–**C**)) and patient (panels (**D**–**F**)) classification performance by CINE images for all models measured with ROC curves. ROC AUC—Area under Receiver Operating Characteristic Curve, Sens.—Sensitivity, Spec.—Specificity.

Expectedly, the imaging protocol had an important effect on the diagnostic accuracy for all considered deep learning techniques and prediction protocols. Independent of the deep learning technique, LGE-trained models achieved the best diagnostic performance (Figure 1). The absolute best performance was observed with the fine-tuning deep learning technique, with the ROC AUC score of 0.96, resulting in 94% sensitivity and 90% specificity, respectively. Second best was a fine-tuned model trained on MOLLI images, with the best ROC AUC score of 0.93, and the diagnostic accuracy of 91% and 82%. A detailed performance of MOLLI images classification is depicted in Figure 2. CINE images were the hardest to classify (ROC AUC 0.89–0.91, for all deep learning techniques), as exemplified in Figure 3. The best diagnostic accuracy was achieved with a fine-tuned model, with 85% sensitivity and 86% specificity.

### 3.4. The Effect of the Deep Learning Technique on Diagnostic Accuracy

All three modeling techniques—feature extraction, from scratch, and fine-tuning—achieved high ROC AUC scores (0.89–0.96) for patient classification. The best diagnostic accuracy, in terms of sensitivity and specificity, was always obtained with the fine-tuning transfer technique (Table 2). From scratch and fine-tuning had comparable mean ROC AUC scores for all imaging protocols; however, the fine-tuning technique had a better diagnostic accuracy (sensitivity range 0.85–0.94 vs. 0.84–0.91). While the performance of the feature extraction technique, in terms of the mean ROC AUC score, stayed on par with the two other techniques, this technique recorded the lowest diagnostic accuracy performance for all imaging protocols (sensitivity range 0.77–0.97). For instance, LGE and MOLLI imaging protocols showed the closest performance in terms of the mean ROC AUC score. While both feature-extraction and fine-tuning techniques achieved comparable ROC AUC scores of 0.92 and 0.93, respectively, the fine-tuning model had a better operating point of 91%, 82% (sensitivity, specificity) vs. 85%, 86% for feature extraction.

### 3.5. The Effect of Prediction Protocol on Diagnostic Accuracy

In all cases, patient classification outperformed image prediction. In particular, it boosted the ROC AUC score for all imaging protocols of the feature extraction technique by 8–10%, by 4–5% for the “from-scratch” technique, and by 2–3% for the fine-tuning technique.

## 4. Discussion

Although LGE CMR imaging represents a real alternative to myocardial biopsy for diagnosing CA with an excellent diagnostic accuracy [19], readers in CMR centers with a low volume of referrals for the detection of myocardial storage diseases or a low volume of cardiac CMRs in general may overlook nonspecific or rare signs for CA. In light of the high prevalence of the disease and emerging therapeutic options [10], we feel that there is an urgent need to avoid lacking diagnoses with regard to CA. Herein, inspired by the hugely successful applications of state-of-the-art deep learning techniques in image understanding [13] and particularly transfer learning techniques in the medical imaging domain [14], we used CNNs to develop a fully automated algorithm for the diagnosis of CA using CMR. We were able to achieve highly accurate (average ROC AUC scores 0.90–0.96) fully automated CA prediction models validated on a cohort of 502 patients (*n* = 82 positive CA patients with EMB ground-truth labels).

### 4.1. Cardiac Magnetic Resonance Imaging for the Diagnosis of Cardiac Amyloidosis

According to Chacko et al. [20] and Fontana et al. [21], CMR should always be used if there is a suspicion of CA, because morphological changes in CMR are clearly visible. Indeed, after the administration of an extrinsic gadolinium-based contrast agent, CMR imaging can reveal characteristic LGE patterns alongside other morphological features, such as myocardial thickening, atrial dilatation, and pericardial and/or pleural effusions. It is furthermore possible to visually determine CA-specific gadolinium kinetics, such as faster washout of gadolinium from myocardium, and blood pool when compared with that of nonamyloid control subjects [22]. In addition, T1 mapping methods allow one to measure this abnormal gadolinium kinetics [23]. In fact, Gillmore at al. published a comprehensive algorithm for the diagnostic work-up of CA, which includes CMR as one baseline diagnostic modality [8]. Previously, Austin et al. [24] concluded that LGE-CMR was the most accurate noninvasive predictor of EMB-positive CA, with sensitivity, specificity, and positive and negative predictive values of 88%, 95%, 93%, and 90%, respectively. Similarly, Bhatti et al. [25,26] proposed a CMR pattern, which they applied to 251 CA patients (63 ± 10 years, 36% females), and achieved a sensitivity and NPV of 100%, and an ROC AUC score of 0.9.

However, there is evidence that in certain cases, CMR may not be enough to establish a reliable CA diagnosis. For the diagnosis of TTR CA, the most meaningful test presently is DPD bone scintigraphy. At the same time, in AL CA, DPD bone scintigraphy is not reliable and may frequently be completely normal. Furthermore, the signs of AL CA may be nonspecific on CMR scans, and CMR may even appear unremarkable although CA is present [9]. This is important to notice, as AL CA seems to be at least as frequent as TTR but affects younger patients and is characterized by a significantly higher morbidity and mortality than TTR CA [10]. Importantly, however, underlying conditions such as plasma cell dyscrasia (i.e., multiple myeloma) make AL CA an effectively treatable disease today [10]. Thus, particularly for AL CA patients, the diagnosis based on CMR findings is highly relevant.

### 4.2. Role and Contribution of AI for CA Diagnosis

Recently, AI has been successfully used to automate the diagnosis of CA from different data modalities. Goto et al. [27] have proposed two CNN-based prediction models for ECG (CA, *n* = 130) and echocardiography (CA, *n* = 70) data, achieving 0.85 ROC AUC and 0.91 ROC AUC scores, respectively. Our group [28] built a gradient-boosted tree prediction model for routinely available lab parameters (CA, *n* = 121) and achieved an ROC AUC score of 0.86 on the test set. Martini et al. [29] trained a CNN-based prediction model for LGE CMR images (CA, *n* = 107) and achieved an ROC AUC score of 0.982 on the test set. Our work can be directly compared to that of Martini et al., with some notable differences. First, we considered a larger patient cohort (*n* = 502 vs. *n* = 206) and a more realistic CA prevalence in a specialized center of 16% vs. 52%; second, on top of the LGE MRI, we also applied a CNN-based model to other imaging protocols, namely T1 mapping and raw CINE images. Our results confirm that an AI prediction model does not require any advanced knowledge of the disease and can potentially be agnostic of a specific imaging protocol.

These recent results demonstrate a remarkable milestone in an attempt to establish a fully computational diagnostic path for the diagnosis of CA to support the complex diagnostic work-up requiring a profound knowledge of experts from different disciplines. Comparing the performance of AI models on different data modalities, we can see that those that process CMR images are the ones that give the best diagnostic accuracy overall.

In their review, Slomka et al. [30] hypothesized that, due to the precise delineation of myocardial contours in LGE images, fully automatic feature extraction with deep learning techniques should be relatively easy. Our results, as well as those of Martini et al. [29], confirm this hypothesis. Therefore, we believe that at this point, it is almost inevitable that AI is tightly incorporated into a routine CA diagnosis practice. However, the intrinsic and extrinsic problems to AI most likely slow down its adoption rate at cardiac imaging centers. One of the biggest concerns of AI models is their lack of interpretability. Some of the noninvasive diagnostic algorithms rely on a list of accepted radiomic features, such as, shape features and the number of connected voxels that share the same intensity, which is widely accepted in clinical practice across the world. Current AI models do not necessarily form their predictions based on these accepted numeric features. Hopefully, the research on explainable AI (XAI) [31] may soon find an answer by translating low-level patterns recognized by cryptic AI models into the language of accepted radiomic features. Extrinsic to AI, data uniformity, as well as the lack of standardization of data acquisition pipelines, are among the biggest challenges for reusable clinical prediction models [32]. To accelerate a successful adoption of AI into a cardiac clinical practice, solving these intrinsic and extrinsic to AI challenges should be prioritized next.

## 5. Limitations

AI algorithms are known to be data hungry and require significantly more positive samples compared to traditional statistical clinical prediction models [33]. Therefore, to increase the sample size of CA positives, we did not focus on the development of separate patient profiles for AL and ATTR (61% of all CA patients in our dataset). However, there is evidence that transmural and subendocardial LGE patterns may differentiate AL from TTR (10)]. In addition, we observed differences extracellular volume in our dataset (Supplementary Table S1). Therefore, it is our priority for future work to collect representative sample sizes for MR images of both CA types and develop their patient profiles using CNNs. In addition, we would like to test if adding T2 time values as a marker would improve the diagnostic ability of the algorithm.

We are aware that this was a single-center study, and therefore, our developed algorithm may not generalize well to the general population. For example, patients with renal impairment (GFR < 30 mL/min/1.73 m^2^) did not undergo CMR imaging. Patients with CA had elevated Troponin T levels (*p* < 0.001), which is a well-known hallmark of CA [34]. Furthermore, because most CA patients were in advanced HF stages, our current algorithm would most likely fail to identify individuals with early or preclinical disease, which is a clear limitation of this study. However, our findings may fuel future research attempting to perform early diagnosis of CA.

Another limitation of the study is a gender mismatch between patients and controls. While it is known that males have a higher incidence of amyloidosis, which is also reflected in our cohort, we had slightly more female patients among controls.

Lastly, in our control patient cohort, we did not perform EMB for the exclusion of CA. However, control patients had alternative diagnoses with congruence between clinical presentation and imaging.

Notwithstanding such limitations, we firmly believe that prediction systems become more accurate if we are able to increase the available sample size. What we need for further validation studies are more publicly available cardiac datasets, similar to what is happening in the adjacent medical domains [35].

## 6. Conclusions

We demonstrate here that an automated classification of CA patients by CMR images using state-of-the-art CNNs is possible and akin to human experts (ROC AUC 0.96 for LGE CMR). This result likely contributes to the establishment of fully computational diagnostic approaches operating on CMR images for CA. With a future perspective, we firmly believe that our experience and guidelines for algorithmic construction of computational and noninvasive diagnostic tools will support the less experienced CMR centers with a low volume of CA. Our hope is that in future clinical practice, we will be able to avoid EMB altogether and establish an accurate diagnosis of CA with noninvasive techniques, such as CMR imaging, in the earliest stages of this rare disease.

## Figures and Tables

**Table 1:** Baseline characteristics of the study population.

	Non-Amyloidosis Related HF (*n* = 420)	Amyloidosis (*n* = 82)	*p*-Value (Adjusted)
Clinical parameters, median (IQR)			
Age, years	66.0 (50.0–75.0)	75.0 (68.0–82.5)	**<0.001**
Male sex, no. (%)	188 (44.9)	52 (65.8)	**0.003**
Height, cm	169.5 (162.0–178.0)	167.0 (162.5–172.0)	0.578
Weight, kg	76.0 (65.8–88.2)	75.0 (65.0–79.5)	0.596
Body mass index, kg/m^2^	27.0 (23.9–31.0)	25.5 (23.9–28.7)	0.066
Laboratory parameters, median (IQR)			
NT-proBNP, pg/mL	452.0 (143.9–1380.0)	3002.0 (1282.5–7453.0)	**<0.001**
Serum creatinine, mg/dL	0.9 (0.8–1.1)	1.2 (1.0–1.6)	**<0.001**
Estimated GFR, mL/min/1.73 m^2^	78.0 (55.0–106.0)	50.0 (38.8–60.5)	**<0.001**
C-Reactive Protein, mg/dL	0.3 (0.1–0.7)	0.3 (0.2–0.7)	0.340
Troponin T, mg/L	17.0 (7.0–29.0)	79.0 (64.0–122.0)	**<0.001**
NYHA functional class, no. (%)			**<0.001**
I	167 (40.3)	10 (12.8)	
II	125 (30.2)	24 (30.8)	
III	107 (25.8)	40 (51.3)	
IV	13 (3.1)	2 (2.6)	
Missing data	2 (0.5)	2 (2.6)	
Medical history, no. (%)			
Hypertension	304 (72.7)	50 (63.3)	0.236
Atrial fibrillation	127 (30.8)	36 (46.2)	0.031
Coronary artery disease	110 (26.6)	20 (25.3)	0.917
Myocardial infarction	42 (10.2)	4 (5.1)	0.339
Percutaneous coronary intervention	57 (13.7)	8 (10.1)	0.591
Coronary artery bypass grafting	22 (5.3)	5 (6.3)	0.829
Diabetes mellitus type II	77 (18.4)	13 (16.5)	0.829
Treatment, no. (%) *			
Oral anticoagulants	134 (32.4)	41 (52.6)	**0.003**
Diuretic agent	153 (37.0)	50 (64.1)	**<0.001**
Mineralocorticoid-receptor antagonist	99 (24.0)	28 (35.9)	0.084
ACE inhibitor or ARB	229 (55.4)	36 (46.2)	0.278
Beta-blocker	232 (56.2)	36 (46.2)	0.245
Calcium channel antagonist	66 (16.0)	8 (10.3)	0.478
Statin	159 (38.4)	23 (29.5)	0.278
Cardiac magnetic resonance imaging parameters, median (IQR)			
Myocardial native T1 time, ms	1050.9 (998.1–1103.8)	1107.6 (1074.5–1140.7)	**<0.001**
Extracellular volume, %	33.5 (28.8–38.3)	46.7 (40.6–52.8)	**<0.001**

* Medication at the time point of referral to expert center. Values are given as median and interquartile range (IQR) or total numbers and percent. Bold numbers indicate statistical significance with *p*-values < 0.05. HF indicates heart failure; NT-proBNP, *n*- terminal prohormone of brain natriuretic peptide; GFR, glomerular filtration rate; NYHA, New York Heart Association; ACE, angiotensin converting enzyme; and ARB, angiotensin receptor blocker.

**Table 2:** Ten-fold cross-validated performance of prediction models for different imaging protocols.

Imaging Protocol		Feature Extraction		From Scratch		Fine-Tuning	
		ROC AUC	Se (Sp)	ROC AUC	Se (Sp)	ROC AUC	Se (Sp)
LGE	Patient	0.96	0.97 (0.81)	0.95	0.91 (0.91)	0.96	0.94 (0.9)
	Image	0.87	0.8 (0.77)	0.89	0.82 (0.81)	0.93	0.82 (0.88)
MOLLI	Patient	0.92	0.85 (0.86)	0.92	0.86 (0.86)	0.93	0.91 (0.82)
	Image	0.84	0.72 (0.8)	0.88	0.79 (0.82)	0.91	0.8 (0.87)
CINE	Patient	0.91	0.77 (0.95)	0.89	0.84 (0.82)	0.90	0.85 (0.86)
	Image	0.81	0.69 (0.78)	0.84	0.72 (0.83)	0.88	0.78 (0.85)

Note: LGE—late gadolinium enhancement, MOLLI—modified look-locker inversion recovery, Patient—average prediction over all images of a patient, and Image—prediction on one image. ROC AUC scores are averages of 10-fold cross-validation. Sensitivity and specificity are computed from mean receiver operating curves with Youden’s J statistic. The best results are in bold. ROC AUC-Area under Receiver Operating Characteristic Curve, Se-Sensitivity, Sp–Specificity.

## Data Availability

The data underlying this article cannot be shared publicly for privacy reasons of individuals that participated in the study. The data will be shared on reasonable request to the corresponding author.

## Supplementary Materials

The following are available online at https://www.mdpi.com/article/10.3390/jpm11121268/s1, Supplementary Table S1. Cardiac magnetic resonance imaging parameters for AL and ATTR, Supplementary Figure S1 Simplified convolutional neural network architecture for automatic diagnosis of amyloidosis. Figure block (**A**) gives a high-level view of convolutional pipeline-input image is fed through a composition of convolutional blocks (**B**), Supplementary Figure S2 Amyloidosis prediction with feature extraction technique, Supplementary Figure S3 Amyloidosis prediction by fine-tuning a pre-trained convolutional network.

## Abbreviations and Acronyms

CA	cardiac amyloidosis
CMR	cardiac magnetic resonance
HFpEF	heart failure with preserved ejection fraction
HF	heart failure
AL	amyloid light chains
LV	left ventricle
HF	heart failure
CNN	convolutional neural network
ECG	electrocardiogram
EMB	endomyocardial biopsy
LGE	late gadolinium enhancement
MOLLI	modified look-locker inversion recovery
ECV	extracellular volume
CV	cross-validation
ROC AUC	varea under the receiver-operating characteristic curve
NT-proBNP	N-terminal prohormone of brain natriuretic peptide
XAI	explainable artificial intelligence
