# Supplementary material for: Convolutional Neural Networks for Fully Automated Diagnosis of Cardiac Amyloidosis by Cardiac Magnetic Resonance Imaging
Source: J Pers Med. 2021 Dec 1;11(12):1268. doi: 10.3390/jpm11121268 (PMC8705947; doi:10.3390/jpm11121268)
Supplement: Supplementary file 1 [file jpm-11-01268-s001.zip › jpm-1419710-supplementary.pdf]

Supplementary Tables

Supplementary Table S1. Cardiac magnetic resonance imaging parameters for AL and ATTR.

	**AL (n = 33)**	**ATTR (n = 49)**	***p*-values**
**Median (IQR)**			
Myocardial native T1 time, ms	1102.5 (1052.5 – 1152.6)	1112.7 (1069.3 – 1156.0)	0.3
Extracellular volume, %	43.0 (33.7 – 52.4)	49.5 (40.6 – 58.4)	**<0.001**

Convolutional Neural Networks and Transfer Learning

Convolutional Neural Networks (CNN) "learn" relevant features (image characteristics) in a hierarchical manner from raw imaging data (Supplementary Figure S1). They do so by using a convolutional filter architecture that relies on progressive image down-sampling (A). The layered architecture of CNNs models hierarchical compositionality of learned filters – from low-level filters (e.g., local edge and sharp changes detectors) to high-level semantic filters. Successful training of CNNs requires large data sets. Transfer learning (B), a technique that reuses the information learned on a different dataset, is used to alleviate problems of small data sets. Transfer learning is based on feature extraction (Supplementary Figure S2) and fine-tuning (Supplementary Figure S3). By feature extraction image / data characteristics are filtered out without changing the configuration of the filters (weights) inside the network. Essentially, a CNN acts as a big filter that ejects a feature representation for each input image. By fine-tuning the configuration of weights is "adjusted" in a controlled way. This can be done by freezing lower layers (very basic patterns) and updating weights for higher layers (Supplementary Figure S3). Fine-tuning a network may result in newly learned filters in the upper layers.

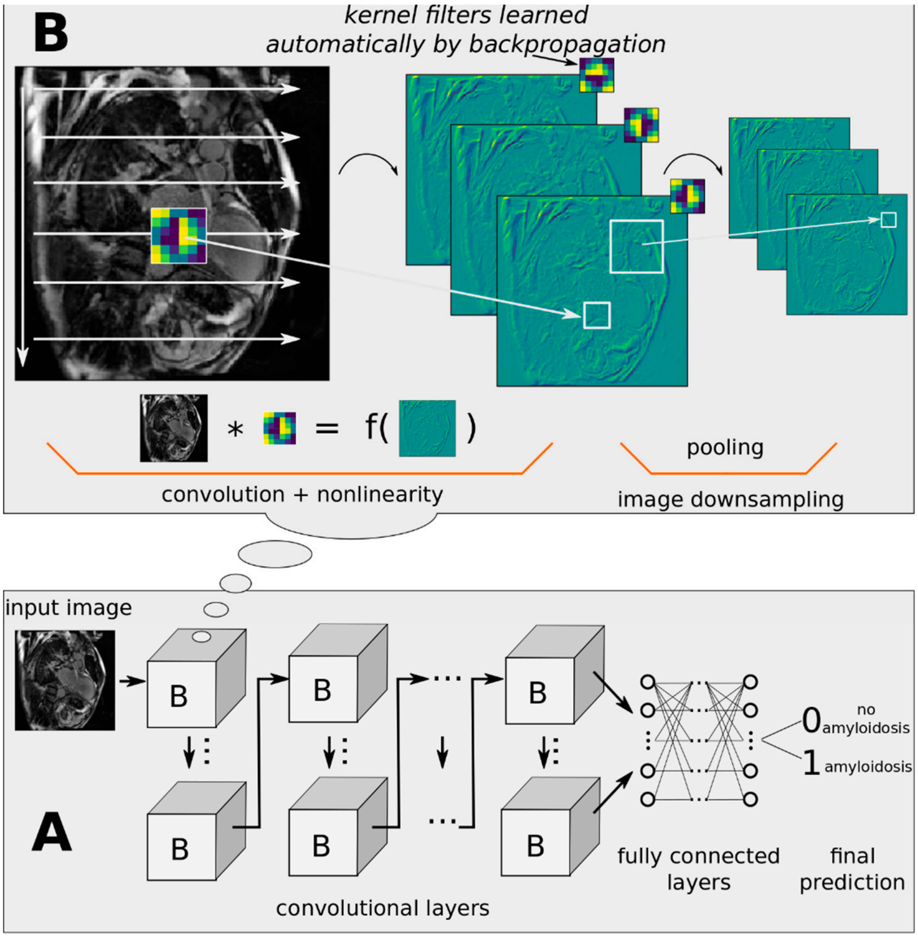

Supplementary Figure S1 Simplified convolutional neural network architecture for automatic diagnosis of amyloidosis. Figure block A gives a high-level view of convolutional pipeline - input image is fed through a composition of convolutional blocks (B). Each block acts as a convolutional filter, followed by a non-linear projection and a pooling layer. Kernel of a convolutional block (receptive field) is learned from raw image data through the process of backpropagation. Final convolutional layers are aggregated and fed through fully connected layers that act as a binary classifier.

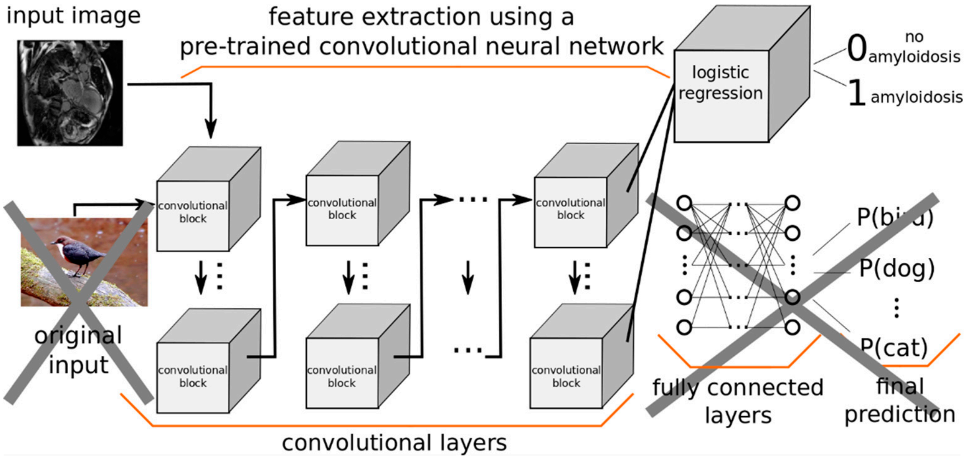

Supplementary Figure S2 Amyloidosis prediction with feature extraction technique. A convolutional neural network trained on a large image dataset (not necessarily related to images of heart) is used as a feature extractor for raw MR images. These features are then used as input to a logistic regression for binary prediction.

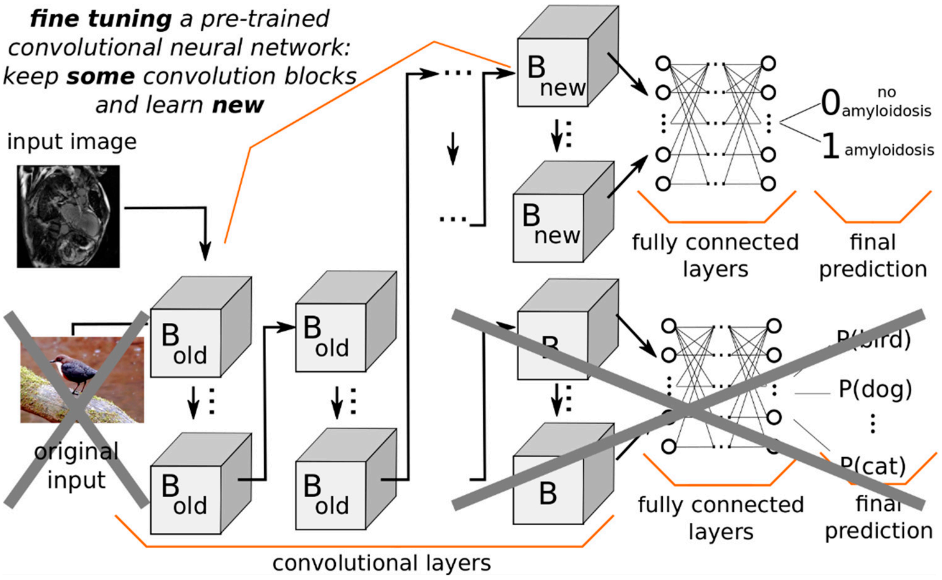

Supplementary Figure S3 Amyloidosis prediction by fine-tuning a pre-trained convolutional network. In contrast to feature extraction, the weight configuration of a pre-trained network is changed through backpropagation – a network builds on general patterns and learns new fine-tuned ones.

References

1. Krizhevsky A, Sutskever I, Hinton GE. ImageNet classification with deep convolutional neural networks. Proceedings of the 25th International Conference on Neural Information Processing Systems - Volume 1. Lake Tahoe, Nevada: Curran Associates Inc., 2012:1097–1105.
2. Litjens G, Kooi T, Bejnordi BE et al. A survey on deep learning in medical image analysis. Med Image Anal 2017;42:60-88.
